# Two *Bacillus* PGPB Strains in Wheat and Soybean: Wheat Growth Promotion Without Detectable Rhizosphere Microbiome Restructuring

**DOI:** 10.3390/ijms27177873

**Published:** 2026-09-03

**Authors:** Elena Nikolaevna Voronina, Ekaterina Alexeevna Sokolova, Irina Nikolaevna Tromenschleger, Olga Viktorovna Mishukova, Valeria Aleksandrovna Fedorets, Inna Viktorovna Khlistun, Oleg Aleksandrovich Savenkov, Oleg Igorevich Saprikin, Maria Dmitrievna Buyanova, Irina Mikhailovna Filippova, Marina Andreevna Glukhova, Evgeny Ivanovich Rogaev, Lada Vladimirovna Zhohova, Andrey Dmitrievich Manakhov, Natalya Valentinovna Smirnova

**Affiliations:** 1 V. A. Knorre Institute of Chemical Biology and Fundamental Medicine, Siberian Branch of the Russian Academy of Sciences (ICBFM SB RAS), 630090 Novosibirsk, Russia; sokolova_ea@1bio.ru (E.A.S.); fedorets@1bio.ru (V.A.F.);; 2 Institute of Soil Science and Agrochemistry, Siberian Branch of Russian Academy of Sciences, 630090 Novosibirsk, Russianvsmirnova29@gmail.com (N.V.S.); 3 Center for Genetics and Life Science, Sirius University of Science and Technology, 354340 Sirius Federal Territory, Russia

**Keywords:** PGPB, *Bacillus*, rhizosphere microbiome, DNRA, plant growth promotion, application count, genotype-phenotype correlation

## Abstract

Plant growth-promoting bacteria (PGPB) are increasingly deployed as biofertilizers, yet the link between an inoculant’s genomic potential and its realized effect on the plant is rarely assessed within an integrative framework that jointly captures the rhizosphere microbiome, plant phenotype, and strain genome. Two *Bacillus* strains—*B. halotolerans* 1453 and *B. pumilus* 630—were applied to wheat and soybean in a factorial pot experiment (2 strains × 2 application methods × 3 frequencies + control, 3–4 replicates). Rhizosphere samples (*n* = 67 after filtering) were profiled by 16S rRNA sequencing with PICRUSt2 functional prediction and compositional validation (Aitchison PERMANOVA, ALDEx2, ANCOM-BC2). The PGPB gene repertoire was characterized by genome mining (481 marker genes, 14 categories). Wheat phenotype (six traits) and soybean height were analyzed with models appropriate for count data (Negative Binomial and binomial GLMs) for treatment-vs.-control comparisons, and with factorial ANOVA for decomposition into main effects and interactions. Crop identity was the dominant factor shaping both microbiome structure and function (PERMANOVA R^2^ = 14.7% taxonomically and R^2^ = 7.8% functionally, both *p* < 0.001), with biologically meaningful taxonomic differences between wheat and soybean; strain, application count and method had no significant effect on community composition (R^2^ < 4% each), and co-occurrence networks showed no reliable differences between crops once read depth and sample size were controlled for. Despite this neutrality at the microbiome level, inoculation significantly increased wheat spike count (NB-GLM, all 12 treatments vs. control, *p*adj 0.0002–0.031), ear weight, and stem count, with application count the strongest source of variability and a pronounced strain × application count. Strain 1453 outperformed 630 in spike count (+23.1%, *p* = 0.012) and ear weight (+20.4%, *p* = 0.023); we hypothesize that this may be related to its more complete DNRA pathway (*narGHI* + *nirB*-*nirD*) and biocontrol genes (*bacE*, *srfAA*). Strain 630 produced a less pronounced effect than strain 1453 but was subject to smaller fluctuations across replicates (CV ≈ 16–21% vs. ≈24–26% for 1453), which may reflect better resilience to environmental fluctuations, possibly due to its confirmed *rsbV*/*rsbW* stress-tolerance regulon. Rhizosphere microbiome composition differed clearly by crop (wheat vs. soybean) but showed no detectable response to strain, application method, or application count. Despite this lack of a microbiome signal, inoculation significantly increased wheat spike count and ear weight, with the magnitude and stability of this effect differing by strain. We hypothesize that this strain-dependent difference relates to underlying genomic differences—particularly in nitrogen metabolism (DNRA pathway) and stress-tolerance genes—though this link has not been tested directly and remains a hypothesis for future work.

## 1. Introduction

Plant growth-promoting bacteria (PGPB) enhance crop productivity through a range of mechanisms—nitrogen fixation, phosphate solubilization, phytohormone production, pathogen biocontrol, and induction of stress tolerance. *Bacillus* is among the most widely used PGPB genera in commercial biofertilizers owing to its sporulation capacity, production of antimicrobial lipopeptides, and rhizosphere colonization ability. Despite decades of research and a growing biofertilizer market, field efficacy remains highly inconsistent: the same strains perform well under some conditions and fail under others, and the mechanism underlying successful cases is rarely established directly within a given experiment.

The rhizosphere microbiome is shaped primarily by host plant identity: crop-specific root exudate profiles select for distinct bacterial communities [1,2]. Functional redundancy—the capacity of different taxa to perform equivalent metabolic functions—is a hallmark of rhizosphere microbiomes and may buffer them against perturbation by inoculants [3]. This raises a fundamental question: if the rhizosphere microbiome is functionally redundant and host-determined, how do PGPB inoculants exert a growth-promoting effect at all, and which of the competing mechanisms is responsible?

At least three non-mutually exclusive mechanisms have been proposed. The first, historically dominant in the field, is modification of the resident microbial community: the inoculant displaces pathogens, enhances beneficial commensals, or otherwise restructures rhizosphere community composition and function, and it is this restructuring that mediates the benefit to the plant [4]. The second is direct physiological/biochemical interaction between the inoculant and the plant: nitrogen and phosphorus provisioning [5], hormonal signaling (auxins, gibberellins), iron delivery via siderophores [6], and modulation of ethylene signaling through ACC deaminase [7]—effects that need not entail any detectable change in community composition. The third mechanism is induction of systemic resistance (ISR), in which the inoculant activates the plant’s immune system with little detectable contribution from the microbial community itself [8]. Disentangling these mechanisms requires an integrative approach that jointly captures microbiome state, plant phenotype, and strain genomic potential.

*Bacillus* was selected as the focal genus for this study because it is among the most extensively documented plant growth-promoting rhizobacteria, with reported activity across a wide range of crops and mechanisms [9]. A single *Bacillus* strain can combine several plant-beneficial traits—phosphate solubilization, phytohormone and siderophore production, and antagonism against fungal and bacterial phytopathogens through antimicrobial lipopeptides—together with induced tolerance to abiotic stress [10]. Its capacity to form endospores confers resistance to desiccation and long-term viability outside the host, a practical property that already underlies several commercial *Bacillus*-based biofertilizers and biofungicides. These combined properties make *Bacillus* both agronomically relevant and experimentally tractable for testing whether strain-level differences translate into differences in plant growth-promoting effect.

The two strains used in this study belong to species that differ considerably in how well they have been characterized as PGPB. *B. pumilus* strains have been repeatedly shown to solubilize phosphate, produce siderophores and IAA, fix nitrogen, and tolerate abiotic stress across diverse hosts and soils, and recent comparative genomics has begun to trace this trait variation to specific loci, including structural variants within the bacillibactin biosynthesis cluster [11]. *B. halotolerans*, by contrast, remains comparatively understudied, but whole-genome sequencing of an endophytic, biocontrol-active isolate has identified a core biosynthetic gene set—surfactin, fengycin, bacillaene, bacilysin, bacillibactin, and subtilosin A—as the basis of its antifungal antagonism [12].

We designed this study to test whether inoculation produces a detectable shift in rhizosphere microbiome structure or function, and whether any resulting effect on plant phenotype depends on such a shift or can occur independently of it. We also examined how application method and count interact with strain genomic potential, since dose, timing, and method all affect field performance and are rarely tested together in a single factorial design.

## 2. Results

### 2.1. Rhizosphere Metagenome Analysis: Taxonomy, Function, Inoculant Detection

#### 2.1.1. Taxonomic Differences Between Wheat and Soybean

**Figure 1:**
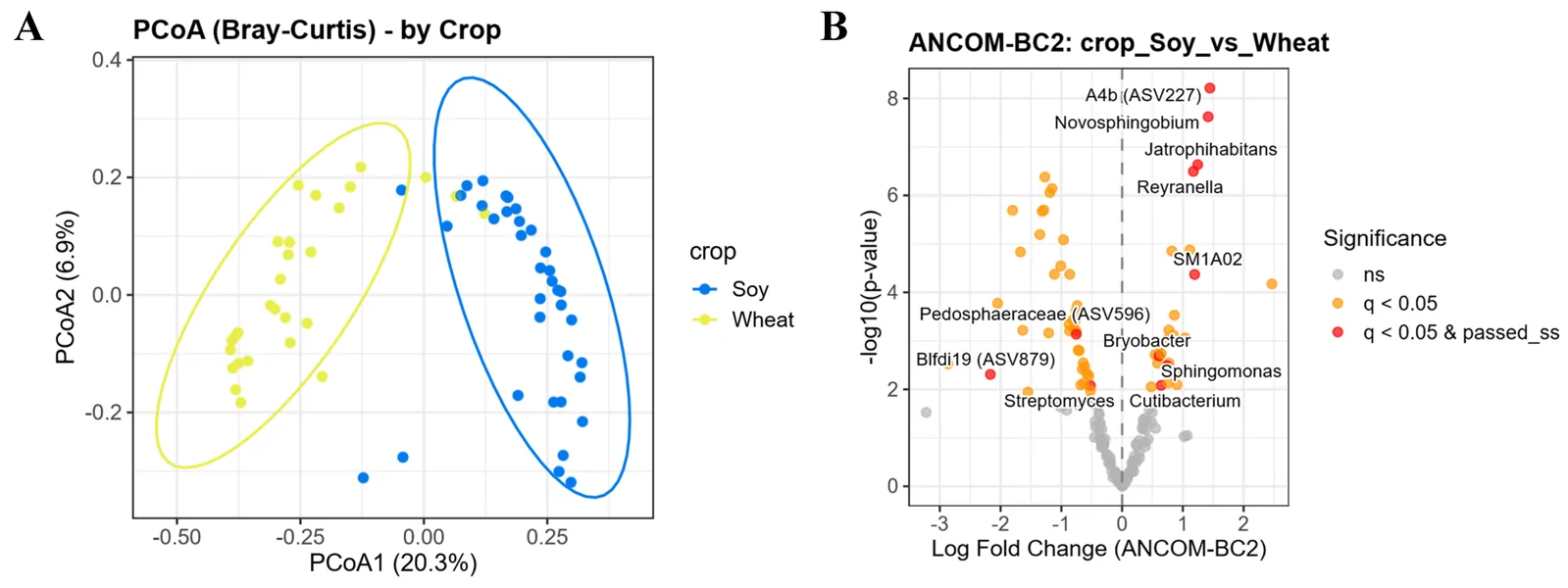
Crop-driven differentiation of the rhizosphere microbiome. (**A**) PCoA Bray–Curtis showing wheat vs. soy separation (PERMANOVA R^2^ = 14.7%, *p* = 0.0001). (**B**) ANCOM-BC2 volcano plot for the crop contrast.

Alpha diversity did not differ between treatments for any index (Kruskal–Wallis *p* > 0.05). Beta diversity, in contrast, differed significantly between crops (Table 1, Figure 1A): crop identity explained 14.7% of Bray–Curtis dissimilarity (F = 10.68, *p* = 0.0001), substantially exceeding strain (R^2^ = 1.6%, *p* = 0.206), method (R^2^ = 1.1%, *p* = 0.762), and application count (R^2^ = 3.7%, *p* = 0.058, trend); a qualitatively similar pattern was observed using Jaccard dissimilarity (R^2^ = 9.3%), indicating that the crop effect was not driven by abundance-weighting alone.

Read depth differed significantly between crops (median 402 for wheat, 3062 for soybean; Mann–Whitney *p* = 1.3 × 10^−11^) and correlated with PCoA axis 1 (Spearman ρ = 0.83). To verify the robustness of crop separation to normalization and depth, PCoA and PERMANOVA were repeated after rarefaction to 150 reads, CSS normalization (metagenomeSeq), DESeq2 VST, and CLR transformation (Aitchison distance). The crop effect remained significant under all methods (PERMANOVA *p* = 0.001). Because dispersions were heterogeneous by crop (see Section 4.3) and depth correlated with PCoA axis 1, the crop effect was further isolated by covariate-adjusted PERMANOVA and db-RDA: conditioning on read depth reduced the crop R^2^ from 14.7% to 10.7% (partial PERMANOVA) and to 9.5% (db-RDA, adjusted R^2^), but crop identity remained highly significant in every ordering (*p* < 0.0001)—indicating partial, not complete, confounding between crop and depth, with a substantial depth-independent crop effect. This means that 14.7% can be considered an upper-bound estimate of the effect on crops. The 9.5–10.7%, adjusted for depth, is a more conservative and more reasonable number for the same difference.

These differences are reproducible at the level of individual taxa and are biologically meaningful. ANCOM-BC2 identified 11 genera significant for crop, versus 0–1 for strain/method/count individually (Figure 1B). Soybean was enriched in *Reyranella* (log2FC = 7.08), *Novosphingobium* (q = 2.3 × 10^−6^), *Jatrophihabitans*, *Bryobacter*, and *Sphingomonas*—taxa characteristic of legume rhizospheres and often associated with diazotrophic activity and phytohormone production; wheat was enriched in *Streptomyces* (q = 0.027, a well-known biocontrol genus, antagonistic to *Fusarium/Rhizoctonia*) and members of Pedosphaeraceae. After the conservative Benjamini–Hochberg correction of DESeq2, only *Reyranella* retains strict significance (*p*adj = 0.009)—but the remaining 10 ANCOM-BC2 genera represent a robust trend, reproducible by two independent methods (ANCOM-BC2, partly LEfSe), rather than random noise, and are biologically consistent with the expected biology of legume (soybean) and cereal (wheat) rhizospheres.

#### 2.1.2. Functional Profile: Compositional Validation

**Figure 2:**
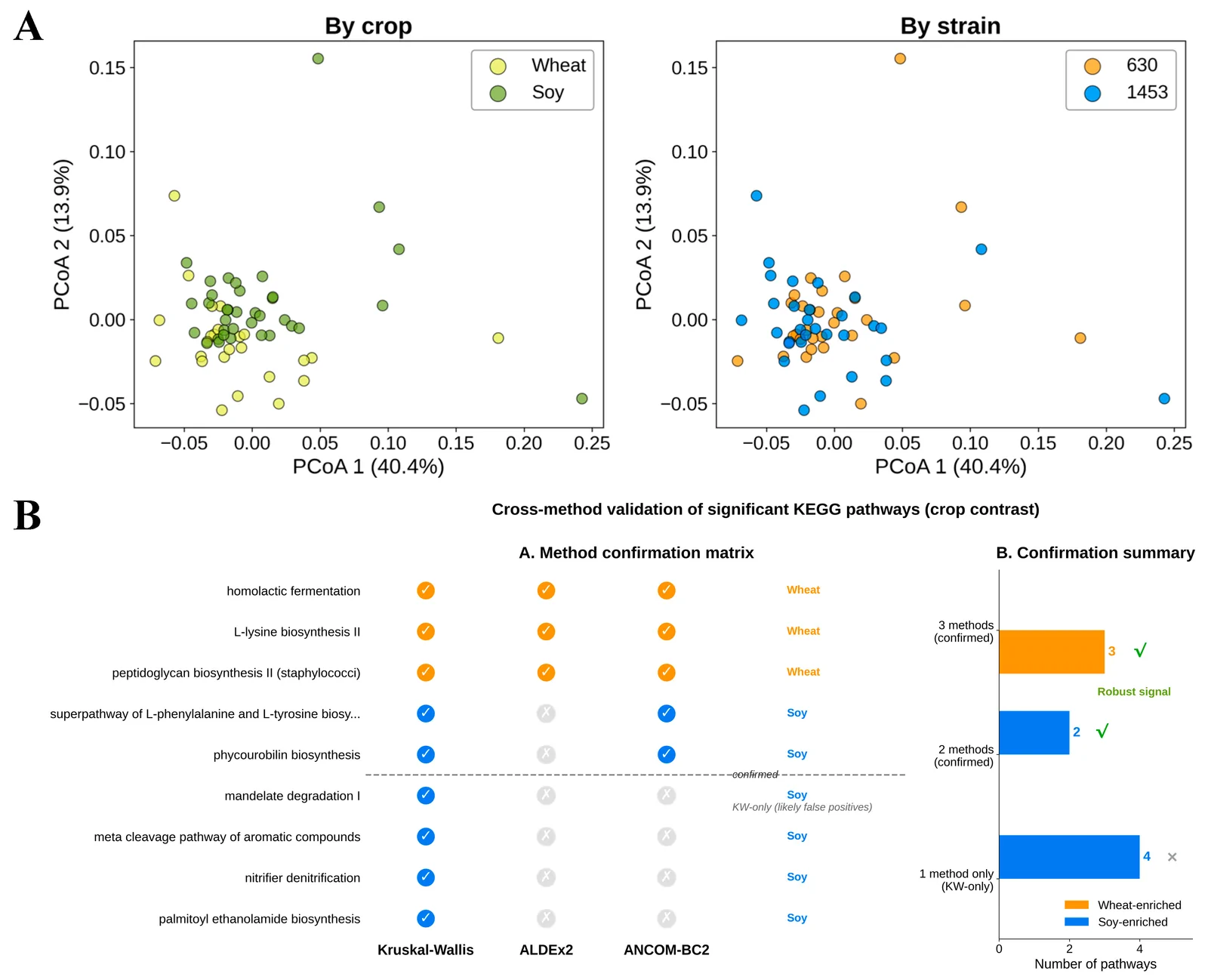
Functional redundancy of the rhizosphere microbiome across crops. (**A**) PICRUSt2 pathway PCoA (CLR-normalized). (**B**) Confirmation matrix: nine paths significant according to Kruskal–Wallis (*p*adj < 0.05) were verified by two compositional methods—ALDEx2 (Wilcoxon, q < 0.05) and ANCOM-BC2 (q < 0.05). Five paths were confirmed by ≥2 methods (green checkmarks) and attributed to a reliable functional signal; four paths are significant only by KW (gray crosses) and classified as probable false positives. The color of the circle indicates the direction of enrichment: orange—wheat, blue—soybeans. The dotted line separates the confirmed and unconfirmed paths.

Because relative abundance remains compositional data, for which rank-based tests distort effect size, the differential functional analysis was validated by three independent methods of increasing rigor—Kruskal–Wallis on relative abundance, ALDEx2, and ANCOM-BC2 on CLR-transformed data (Table 2). Naive Kruskal–Wallis identified 187/519 (36%) significant pathways and 3721/8186 (45%) significant KOs for crop; the compositional methods produced a more conservative but qualitatively consistent picture: ALDEx2 and ANCOM-BC2 confirmed 66 and 79 significant pathways, respectively (KW overstated the number of significant pathways by 2.4–2.8-fold). PERMANOVA on Aitchison distance gave R^2^ = 0.078 (*p* = 0.0001)—higher than on Bray–Curtis (R^2^ = 0.055)—indicating that moving to a strictly compositional method strengthens, rather than weakens, the crop signal (Figure 2A). No pathway/KO/EC was significantly associated with strain, method, or count by any of the three methods.

Three pathways confirmed by all three methods and enriched in wheat represent the most robust functional signal: peptidoglycan biosynthesis II (staphylococci), homolactic fermentation, and L-lysine biosynthesis II. Two pathways enriched in soybean were confirmed by two of the three methods: the superpathway of L-Phe/L-Tyr biosynthesis and the phycourobilin biosynthesis (Figure 2B). Four other originally reported ‘soybean’ pathways were significant only by KW and were not confirmed by the compositional methods—likely false positives, excluded from interpretation.

#### 2.1.3. Detection of Inoculant Strains

Strain 630 was detected at 100% identity in 1 of 30 treated samples (28 reads at a sample depth of 25,288—the highest in the dataset); strain 1453 was not detected in any sample (0/31). A binomial detection model showed that, at the median wheat depth (402 reads), detection probability at 0.1% abundance was only 33.1%, and at 0.01% abundance, only 3.9%. Monte Carlo simulation showed that the observed 1/30 and 0/31 detections are fully compatible with colonization at 10–25% of samples at low abundance; the occupancy model for strain 630 statistically favored the hypothesis of ubiquitous presence at the detection limit (ΔAIC = 2.0) over true absence. On the basis of the available data, inoculant establishment can therefore be neither confirmed nor ruled out—insufficient detection power, rather than absence of an effect, most plausibly explains the negative tracing result.

### 2.2. Phenotypic Effect of Inoculation, Application Count, and Grain Elemental Composition

#### 2.2.1. Inoculation Effects on Wheat Phenotype at Harvest

**Figure 3:**
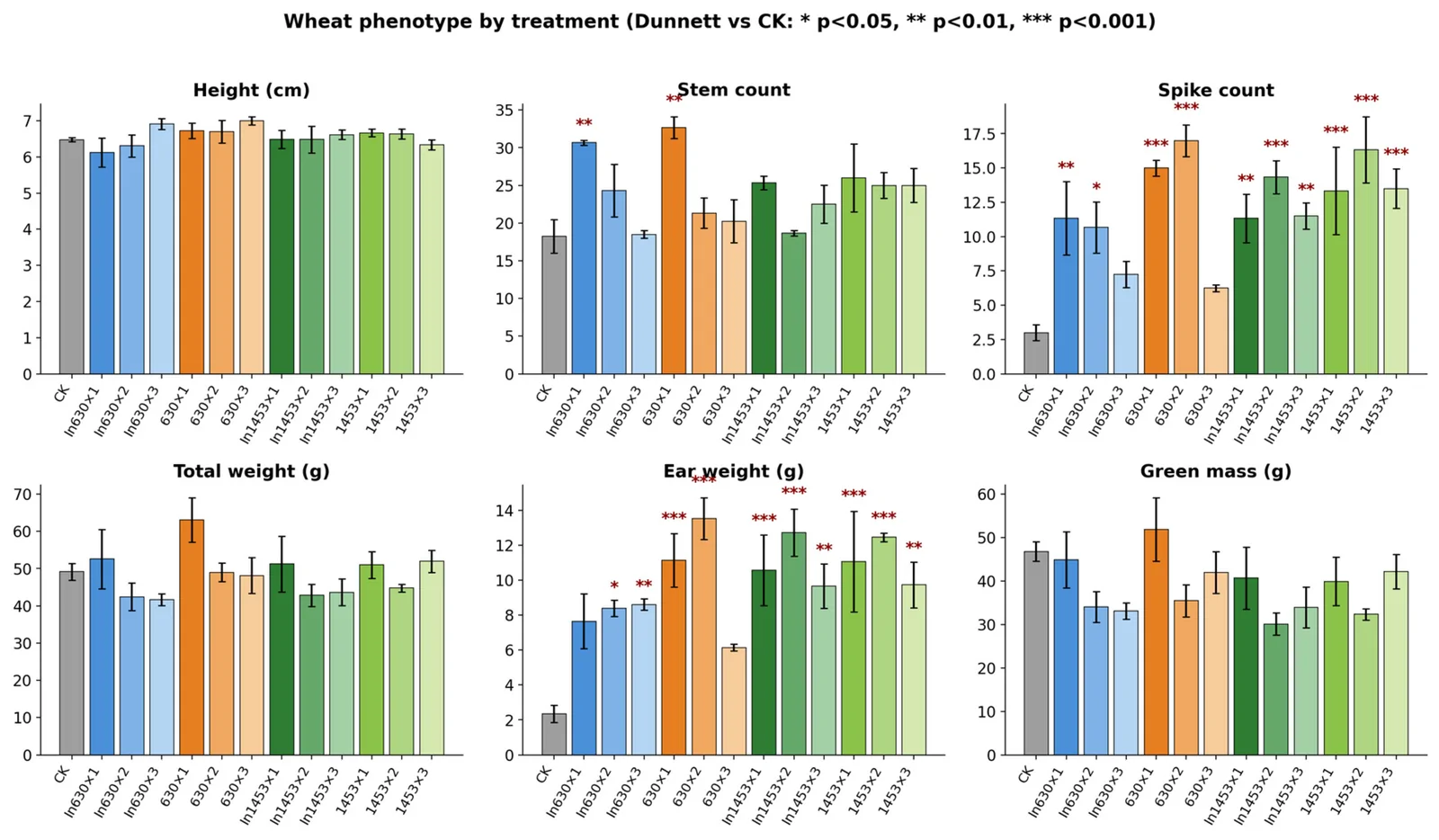
Wheat phenotype response to inoculation treatments. Six-panel boxplot of wheat traits (height, stems, spikes, total weight, ear weight, green mass) across 13 treatments with replicate points and Dunnett significance.

At 10 days after sowing, prior to divergence of the feeding schedules, seedling height did not differ by any factor (*p* > 0.05), ruling out pre-existing heterogeneity of the planting material as a source of subsequent differences. This absence of early divergence stands in contrast to the pronounced treatment effects that had emerged by harvest, which we assessed across all six measured traits (Figure 3).

As shown in Figure 3, reproductive traits responded most consistently: ear weight increased significantly in 10 of 12 treatments (Dunnett vs. CK, *p*adj < 0.05), with only I630 and L630x3 not reaching significance. Stem count increased significantly in only 2 of 12 treatments—I630 and L630, both single-application variants of strain 630—while none of the strain-1453 treatments or higher-frequency 630 treatments reached significance. Total weight and green mass showed no significant differences from the control at the individual-treatment level (Dunnett, all *p*adj > 0.05), despite the trend toward lower green mass at higher frequencies noted in Section 2.2.2. Height was unaffected throughout (all *p*adj = 1).

**Figure 4:**
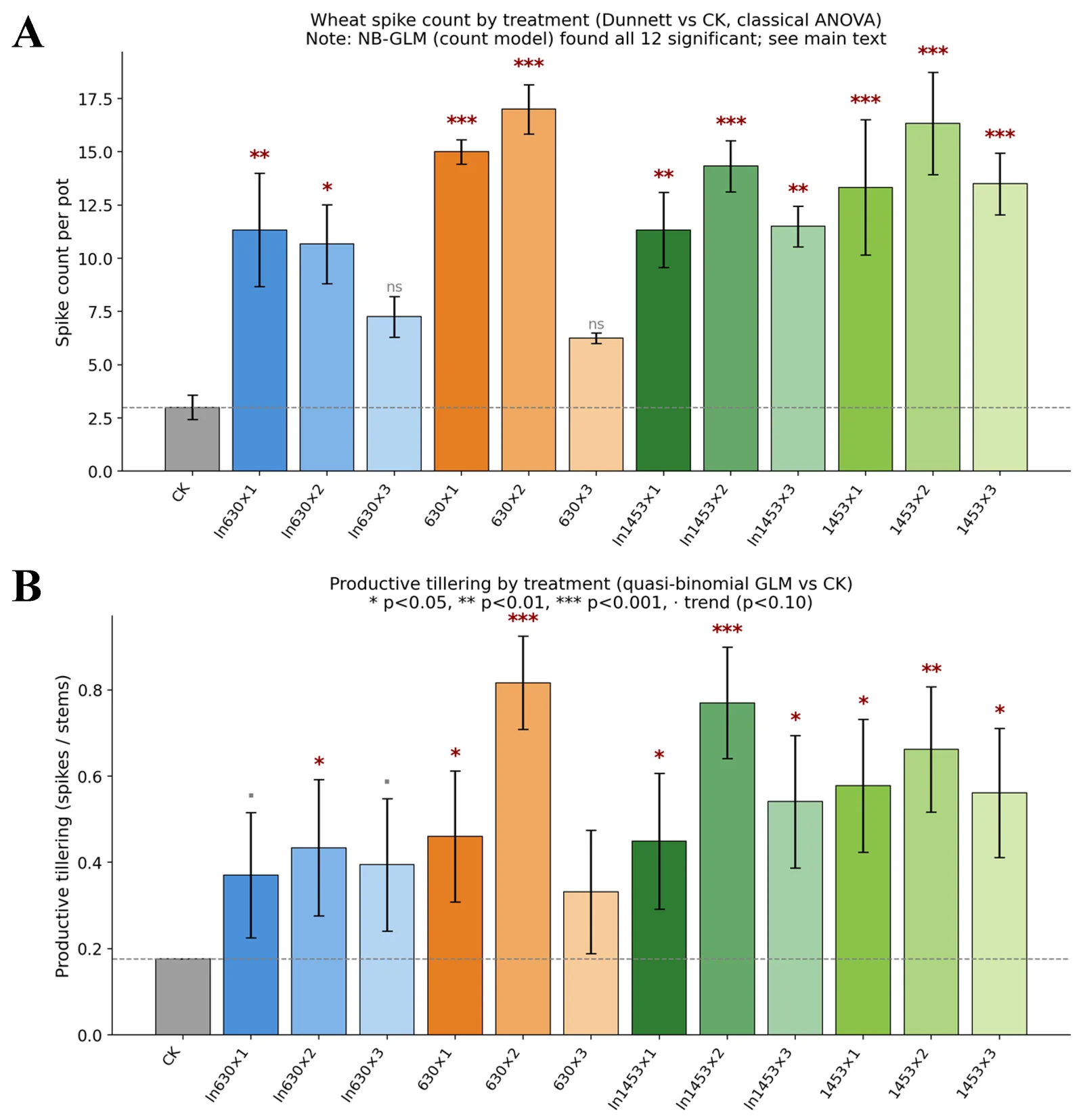
Wheat spike and productive tillering count by inoculation treatment (NB-GLM significance). (**A**) Wheat spike count across 13 treatments (mean ± SEM, individual points). (**B**) Productive tillering (spikes/stems) across 13 treatments (mean ± SEM, individual points).

Among the six traits, spike count showed the most consistent response to inoculation and was therefore analyzed in greater statistical depth using a model appropriate to its count nature. Because spike count is a discrete count variable, the classical Dunnett comparisons shown in Figure 4—which assume approximately normal residuals—are conservative for this trait; we therefore complemented them with a Negative Binomial GLM (NB-GLM) that models the count distribution directly. Modeled this way, the effect of inoculation on spike count proved both highly significant overall and remarkably consistent across treatments: the likelihood-ratio test against the null model was strongly significant (LR = 73.7, df = 12, *p* = 6.5 × 10^−11^), and every one of the 12 inoculated treatments significantly exceeded the control (*p*adj 0.0002–0.031)—spike counts ranged from 6.25 to 17.0, versus just 3.0 for CK, a two- to nearly six-fold increase (Figure 4A).

This gain in spike number translated into a corresponding gain in productive tillering (the proportion of stems that formed a spike): a binomial GLM with quasi-binomial correction confirmed a significant increase for 9 of the 12 treatments (*p*adj 0.019–0.028), with the remaining three showing a consistent trend in the same direction (*p*adj 0.088–0.10) rather than a null result (Figure 4B). Taken together, these two measures indicate that inoculation did not simply increase the number of stems, but shifted a larger share of them toward reproductive output.

#### 2.2.2. Decomposing Treatment Effects: Strain, Application Count and Method

The treatment-vs.-control comparisons above establish that inoculation reliably increases spike count and ear weight, but they do not, on their own, indicate which aspect of the experimental design drives this effect—whether it is the choice of strain, the method of application, how many times the bacteria were applied, or some combination of these. To disentangle these contributions, we decomposed the twelve inoculated treatments (excluding CK) into a full 2 × 2 × 3 factorial ANOVA—strain × method × number of applications (hereafter ‘application count’; modeled as the factor *frequency* with three levels, 1×/2×/4×)—for each of the six wheat traits (Supplementary Table S1).

This decomposition points to application count, rather than strain identity or delivery method, as the primary driver of the phenotypic response: application count was a significant main effect for five of the six traits—stem count (F = 10.98, *p* = 0.0003, η^2^ = 0.44), spike count (F = 10.18, *p* = 0.0005, η^2^ = 0.42), ear weight (F = 6.08, *p* = 0.006, η^2^ = 0.30), total weight (F = 5.30, *p* = 0.011, η^2^ = 0.27), and green mass (F = 5.35, *p* = 0.011, η^2^ = 0.28)—with only height unaffected (*p* = 0.32). Strain identity, by contrast, mattered for a narrower, specifically reproductive set of traits: spike count (F = 7.19, *p* = 0.012, η^2^ = 0.20) and ear weight (F = 5.75, *p* = 0.023, η^2^ = 0.17), in both cases favoring strain 1453. Application method had the most limited main effect, reaching significance only for spike count (F = 6.08, *p* = 0.020, η^2^ = 0.18) and total weight (F = 5.30, *p* = 0.029, η^2^ = 0.16), with drench outperforming seed inoculation.

**Figure 5:**
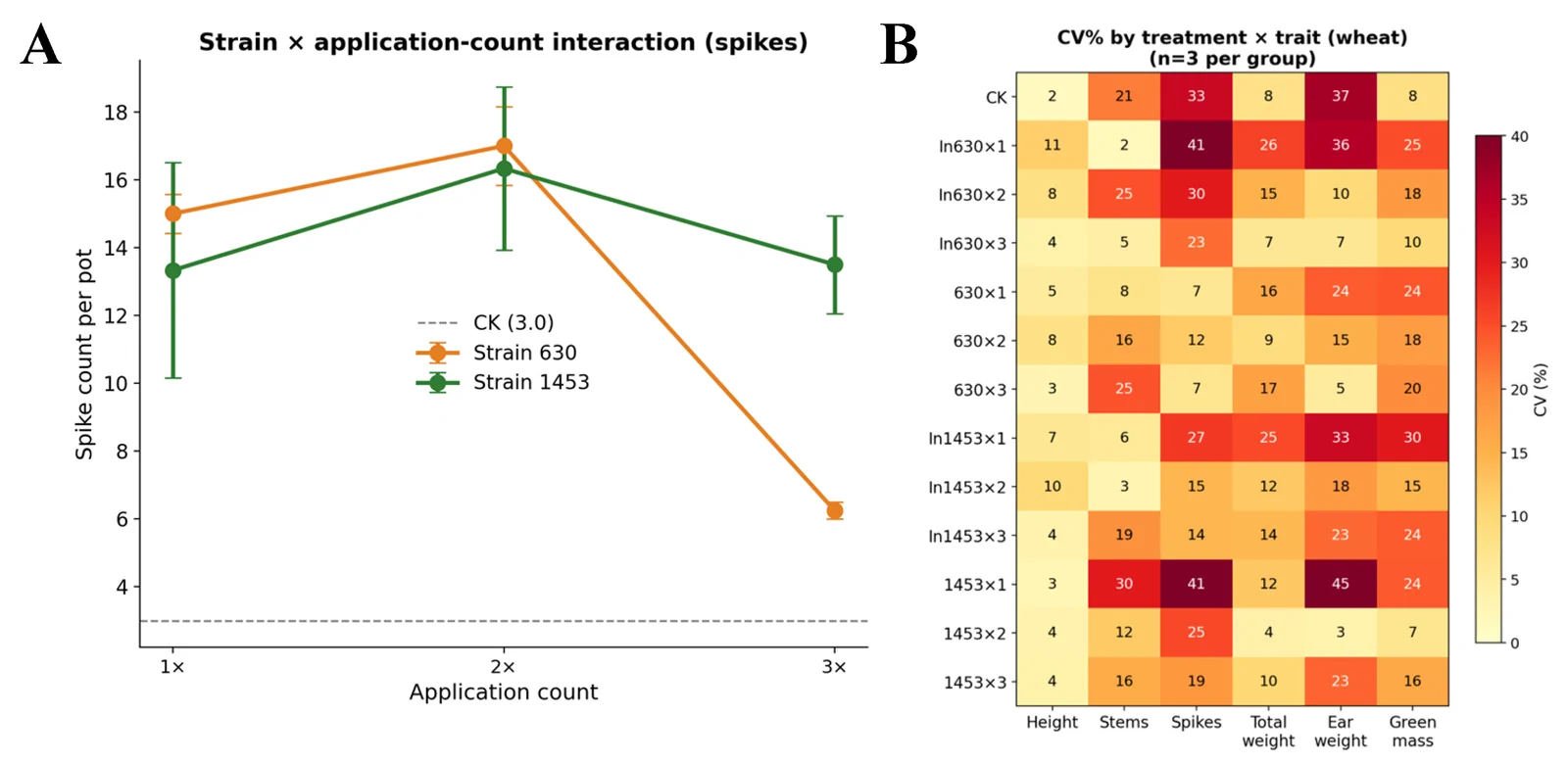
Strain × frequency interaction on wheat spike count and trait variability. (**A**) Strain × frequency interaction plot for spike count with SEM and individual points. (**B**) CV% heatmap for 13 treatments × 6 wheat traits.

Critically, application count did not act independently of strain: the strain × application-count interaction was significant for both stem count (F = 5.25, *p* = 0.012, η^2^ = 0.27) and spike count (F = 4.52, *p* = 0.020, η^2^ = 0.24), indicating that the two strains respond differently as the number of applications increases, rather than simply scaling the same response up or down. This interaction—examined in detail below (Table 3, Figure 5)—turns out to be the more informative result than any single main effect: it is not simply that ‘more applications are better,’ but that the optimal number of applications depends on which strain is applied.

Table 3 makes this pattern concrete for stem count and ear weight. Strain 630 declines steadily as application count increases—stem count falls from 31.7 at 1× to 22.8 at 2× and 19.4 at 4×—whereas strain 1453 remains comparatively stable across the same range (25.7 → 21.8 → 23.8). Ear weight tells a related but distinct story: both strains peak at 2× (630: 9.4 → 11.0 → 7.4; 1453: 10.8 → 12.6 → 9.7), indicating that the two-application regime is not simply ‘safer’ for 630 but genuinely optimal for both strains—what differs by strain is the cost of moving beyond it, not the location of the optimum itself.

Because spike count is a count outcome, the strain × application-count interaction was additionally verified with a distribution-appropriate Poisson GLM (deviance/df = 0.73, indicating adequate fit with no overdispersion; deviance residuals showed no systematic pattern by cell): the interaction remained significant (LR = 9.38, df = 2, *p* = 0.0092). Pairwise contrasts sharpened the picture further: strain 630 declined significantly from 2× to 4× (13.8 → 6.8 spikes, LR = 17.35, *p* < 0.0001), whereas strain 1453 showed no statistically detectable decline over the same range (15.3 → 12.5, LR = 1.99, *p* = 0.158)—a cleaner, model-based contrast than the raw percentage change alone would suggest. Taken together, these results converge on a single conclusion: 2× application is the practical optimum for both strains, but 1453 tolerates deviation from it far better than 630 does.

This asymmetry between the two strains extends beyond the mean response to its variability. Figure 5B summarizes the coefficient of variation (CV%) across all 13 treatments and six traits, and the same 630/1453 contrast reappears here: strain 1453′s higher average productivity comes with greater inter-replicate variability, while strain 630′s more modest response is also the more consistent one—for ear weight, CV was 25.5% in 1453 versus 16.4% in 630, and for spike count, 24.4% versus 20.6%. This pattern indicates that the two strains differ not only in the magnitude of their reproductive effect, but in a productivity–stability trade-off that holds across the full set of treatments, not merely at the 2×/4× contrast highlighted above.

#### 2.2.3. Grain Elemental Composition

Grain elemental composition of wheat (P_2_O_5_, NH_4_, K, Ca, Mg; pooled composite samples, descriptive only, available for 10 of 13 treatments; Table 4) showed lower concentrations of all measured elements, including ammonium-N, in every treated variant relative to the control—consistently across three independent analytical methods (spectrophotometry, potentiometry, AAS). The most plausible explanation is a growth dilution effect: an increase in spike number/mass at a relatively constant total elemental uptake reduces elemental concentration per unit grain mass—i.e., the inoculation effect manifests as enhanced reproductive development rather than increased nutrient density in the grain.

### 2.3. Genomic Differences Between Strains and Their Metabolic Interpretation

#### 2.3.1. Biochemical Characterization

To determine whether genomic potential translates into measurable biochemical output, both strains were characterized by a quantitative in vitro screening panel spanning proline production, growth under osmotic stress, phosphate solubilization, siderophore and gibberellin production, and IAA production (Table 5).

The results reveal a pattern of reciprocal specialization. Strain 630 produced more proline (70.0 vs. 28.9 µg/mL) and retained higher growth under severe osmotic stress (36.9% vs. 15.4% at 15% PEG). Strain 1453, conversely, showed higher phosphate-solubilizing activity (95.3 vs. 55.0 µg/mL), siderophore production (35.6% vs. 19.6%), and gibberellin production (1.56 vs. 0.80 mg/mL).

#### 2.3.2. Genomic Differences Between Strains and Genotype–Phenotype Concordance

**Figure 6:**
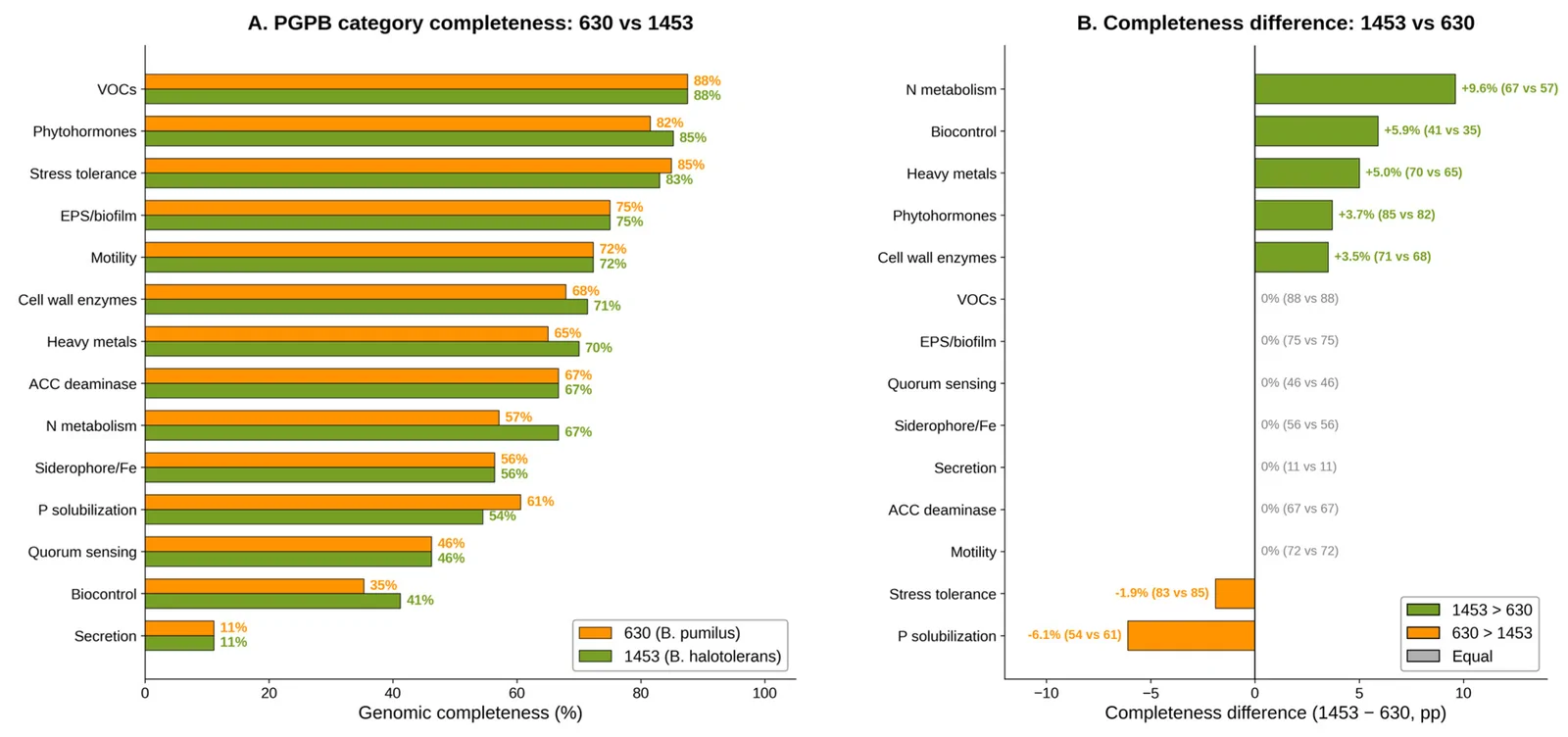
Genomic completeness of PGPB functional categories in strains 630 and 1453. (**A**) Genomic completeness of PGPB categories: 630 vs. 1453. (**B**) PGPB gene category completeness difference between strains 1453 and 630.

Genome mining showed that strains 1453 and 630 share 270 of 481 PGPB marker genes (94–97% of each strain’s complement)—genetically near-identical strains, with 16 genes unique to 1453 and 9 unique to 630, unevenly distributed across the 14 functional categories (Figure 6).

Mapping genomic completeness in these categories onto the corresponding wheat traits yielded no more concordance than chance would predict (11/17 directional mappings, binomial *p* = 0.33). The two clearest individual cases even point in opposite directions: 630′s greater completeness for stress-response genes aligns with its advantage in vegetative growth, whereas its greater completeness for phosphate solubilization does not. Despite 630′s genomic edge in this category, 1453 outperforms it on ear weight—consistent with the in vitro pattern of expression rather than gene content driving that phenotype.

The gene-level data do, however, point to two specific, mechanistically tractable differences between the strains—in nitrogen metabolism and in biocontrol/stress tolerance—which we examine in turn below.

#### 2.3.3. The DNRA Pathway as a Probable Mechanism of Strain-Specific Dose Response

Across the full 481-gene PGPB panel (Table S2), 45 genes scored higher for strain 1453 and 26 scored higher for strain 630 (any score difference ≥ 1); a curated subset of 19 of these genes, selected for a clear mechanistic link to a measured phenotypic trait, was organized into six mechanistic hypotheses (H1–H6, Table S3). Among the six mechanistic hypotheses examined in detail (no formal cross-category ranking test was performed; see Section 4.4), nitrogen metabolism had the most complete combination of confirmed genetic evidence and significant phenotypic support. Neither strain is capable of canonical denitrification (NO_3_^−^ → N_2_): *nirK*/*nirS* and *nosZ* are absent in both. The fate of nitrite is determined by the DNRA pathway (dissimilatory nitrate reduction to ammonium, NO_3_^−^ → NO_2_^−^ → NH_4_^+^): in 1453, *narH* and *narJ* are confirmed (absent in 630), *narG* is confirmed in 1453 (putative in 630), and *nirD* (the small subunit of the NirB-NirD complex, required for NO_2_^−^ → NH_4_^+^ reduction) is likely in 1453 and absent in 630. Without *nirD*, the NirB-NirD complex cannot assemble, and in 630 the DNRA pathway is truncated at the nitrite stage (Table 6).

To rule out alternative nitrite-handling routes that could bypass the NirB-NirD bottleneck, both genomes were additionally screened (BLASTp, profile HMM, tBLASTn; relaxed threshold e-value ≤ 1 × 10^−3^) against the periplasmic *nrfA*/*nrfH* system, the assimilatory reductase *nirA*, and NO-detoxification genes (*hmp*, *norV/W*). *nrfA* and *nrfH*—the specific alternative DNRA route present in some *Bacillus* species [13]—were absent from both genomes by all three methods. NirA was only weakly detected in both strains and, being assimilatory rather than dissimilatory, would not resolve a nitrite bottleneck under nitrate excess even if present; *hmp* and *norV/W* detoxify nitric oxide but do not reduce nitrite to ammonium. No alternative route to complete nitrite reduction was therefore identified in either strain. This screen was targeted at the routes specifically raised as alternatives, and should not be read as an exhaustive survey of all possible nitrite-tolerance mechanisms (e.g., efflux). The two genomes also differ in assembly completeness—100% for 630 versus 94.51% for 1453—so while absence calls in 630 can be made with confidence, the corresponding calls for 1453 are better read as ‘not detected within the current assembly’ rather than as definitive.

This genomic picture is matched by a coherent phenotypic signature. Strain 1453 outperforms 630 by +23.1% in spike count (*p* = 0.012) and +20.4% in ear weight (*p* = 0.023)—both reproductive traits, and both particularly sensitive to nitrogen availability during heading and grain filling. Total weight, by contrast, a measure of vegetative biomass, did not differ between the strains (−2.9%, *p* = 0.575), consistent with nitrogen availability shaping reproductive development specifically rather than overall plant growth. Taken together—confirmed genes, significant and trait-specific phenotypic differences, and a coherent biochemical pathway reconstruction—we assign this hypothesis HIGH confidence.

**Figure 7:**
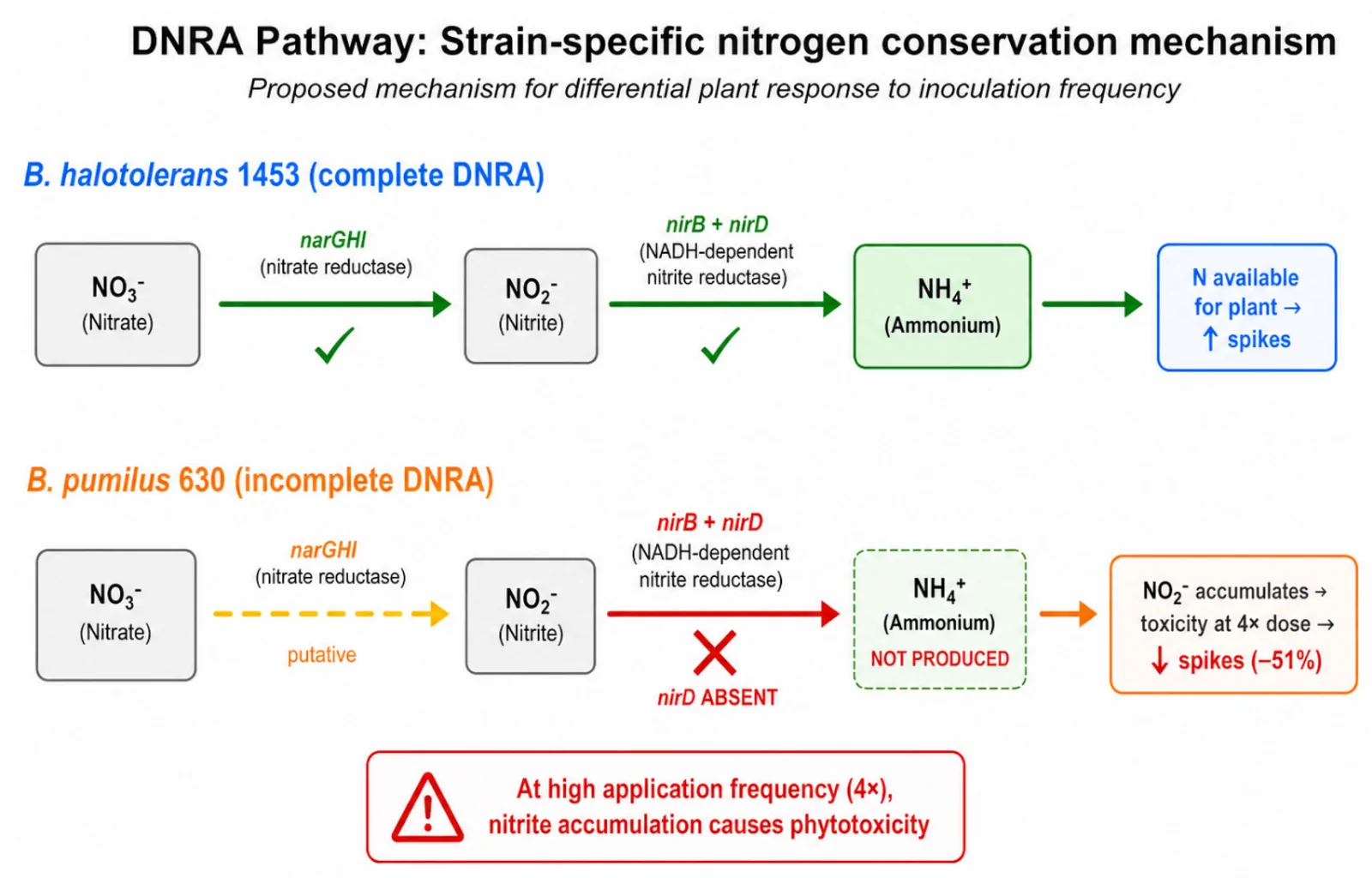
Dissimilatory nitrate reduction to ammonium (DNRA) pathway in strains 1453 and 630. Conceptual schematic of the DNRA pathway in 1453 (complete) versus 630 (incomplete, *nirD* absent).

A working hypothesis explaining the strain × application-count interaction: at a low-to-moderate number of applications (1×, 2×), both strains stimulate heading through shared PGPB mechanisms. At 4× application count, metabolic load increases; 1453, with a complete DNRA pathway, retains nitrogen as NH_4_^+^ and sustains productivity, whereas in 630, nitrite that cannot be further reduced due to the absence of *nirD* may accumulate to phytotoxic concentrations, consistent with the observed decline in spike count at 4×. This is a plausible but experimentally unconfirmed hypothesis: it rests on genomic data and phenotypic patterns rather than direct measurement of nitrite/ammonium concentrations in the rhizosphere (Figure 7).

#### 2.3.4. Biocontrol and Stress Tolerance: A Productivity/Stability Trade-Off

Strain 1453 carries a distinct set of unique biocontrol genes—bacillizin (*bacE*, confirmed), subtilosin (*sboA*, likely), and a stronger surfactin pathway (*srfAA/AB/AD*)—together with extracellular proteases *nprE* (confirmed, unique to 1453) and *nprB* (likely), which mineralize organic nitrogen and could supplement the nitrogen supply captured via the DNRA pathway described above. This genomic profile is consistent with 1453′s higher spike count and ear weight, but it comes with a cost: 1453 also shows greater inter-replicate variability in these same traits, with a CV of ear weight at 25.5% versus 16.4% in 630, and a CV of spike count at 24.4% versus 20.6% (Figure 5B).

Strain 630, by contrast, carries a confirmed general stress-response regulon (*rsbV*, *rsbW* confirmed; *rsbU*, *rsbS* likely) that controls sigma-B-dependent transcription of hundreds of stress genes. This genomic feature aligns with a different phenotypic signature: lower CV in 630 for the reproductive traits, together with its advantage in the vegetative traits green mass (+8.2%) and height (+1.9%). Taken together, these patterns suggest that 630′s stress tolerance supports stable vegetative growth rather than enhancing reproductive output.

The two strains therefore represent complementary strategies rather than one being straightforwardly superior: 1453 delivers higher absolute heading productivity at the cost of greater inter-replicate variability, while 630 delivers a more modest but more reproducible effect.

## 3. Discussion

### 3.1. Differences in the Wheat and Soybean Rhizosphere Microbiome

The consistency of this result across independent methods—beta diversity, differential abundance, and functional prediction after compositional validation—indicates that host-specific selection of wheat and soybean root exudates is the dominant factor structuring rhizosphere community assembly. This is fully consistent with a large body of literature on host-specific recruitment of rhizosphere bacteria across diverse plant taxa—cereals, legumes, solanaceous crops, cotton, maize, and medicinal plants. Emmett et al. [14] showed that rhizosphere community beta diversity correlates positively with host phylogenetic distance, and Matthews et al. [15] found that plant species exert a stronger influence on rhizobacterial community composition than soil type. In our experiment, all samples originated from the same soil, which isolates the crop effect from the soil factor and renders the R^2^ = 14.7% estimate a relatively unconfounded measure of host specificity.

The mechanistic basis of this host specificity is root exudate composition. Sasse et al. [1] formulated the ‘feed your friends’ concept: exudate composition determines which microorganisms gain a competitive advantage in the rhizosphere. Zhalnina et al. [2] showed, in Avena barbata, that exudate chemistry changes predictably over ontogeny, and that bacteria enriched in the rhizosphere have predictable substrate preferences—particularly for aromatic organic acids (nicotinic, shikimic, salicylic, indole-3-acetic); bacteria positively responsive to root growth consumed up to 48% more of these acids than negatively responsive bacteria. Distinct exudate compositions of wheat and soybean (including flavonoids required for the soybean-rhizobium symbiosis) plausibly explain why the two crops assemble distinct microbiomes even in identical soil.

The magnitude of the crop effect varies across studies: Wicaksono et al. [16] reported R^2^ = 50.5% for three medicinal plants from different families (Asteraceae, Solanaceae) with different life forms; Hartman et al. [17] showed that plant identity is the strongest determinant of root microbiome composition, with symbiosis type (mycorrhizal, rhizobial) further modulating diversity. In our study, wheat and soybean are both annual crops grown in identical soil, which is expected to lower R^2^ relative to inter-family comparisons but yields a more precise estimate of the species effect, unconfounded by soil or life-form differences.

For wheat, enrichment of Streptomyces (ANCOM-BC2, q = 0.027) is consistent with the well-known association of streptomycetes with cereal rhizospheres and their role in biocontrol of fungal pathogens; Cangioli et al. [18] showed that wheat cultivar exerts a stronger influence on the rhizosphere community than fertilization regime, and Abdullaeva et al. [19] and Fang et al. [20] showed that wheat domestication weakened natural bacterial biocontrol against fungal pathogens, making the presence of Streptomyces agronomically meaningful rather than a purely taxonomic observation. For soybean, enrichment of *Reyranella*, *Novosphingobium*, *Jatrophihabitans*, *Bryobacter*, and *Sphingomonas* is consistent with the legume rhizosphere’s role as a habitat for diazotrophic and phytohormone-producing taxa; Han et al. [21] showed that specific soybean rhizosphere microorganisms correlate with *Bradyrhizobium/Sinorhizobium* composition in nodules, and Sohn et al. [22] showed that *Bradyrhizobium* dominates nodules at all growth stages. That only *Reyranella* retains significance after the most conservative correction (DESeq2) does not devalue the remaining 10 ANCOM-BC2 genera—this is an expected consequence of differing method conservativeness rather than evidence of no effect: the direction and biological plausibility of these taxa are consistent across methods and with independent literature.

### 3.2. Microbiome-Phenotype Dissociation and Functional Redundancy

Against a backdrop of pronounced crop-specific differences, one contrast stands out: neither strain, method, nor frequency had any significant effect on microbiome structure or function (PERMANOVA R^2^ < 4% for each factor; no pathway, KO, or EC significantly associated with strain/method/frequency by any of the three methods; co-occurrence networks likewise showed no differences once depth/sample size were controlled for)—yet inoculation produced a pronounced, statistically robust effect on wheat reproductive development. Louca et al. [3], in a foundational synthesis, defined functional redundancy as an inevitable property of open microbial systems: many taxonomically distinct organisms can perform the same metabolic function, such that taxonomic and functional community composition are often ‘decoupled’—hundreds of hydrogen-oxidizing microorganisms coexist in groundwater, and dozens of genomes are capable of complete denitrification within a single aquifer.

Our result—crop identity explains a markedly smaller share of variance in the community’s functional profile (7.8%) than in its taxonomic composition (14.7%)—indicates that a substantial portion of the taxonomic crop effect does not translate into functional differences. This is consistent with direct empirical evidence from Castellano-Hinojosa & Strauss [23], who examined 5842 rhizosphere samples across six crop species and asked which taxa and which functions are shared across all of them—the ‘core’ in each case. They found that the taxonomic core (the set of bacterial taxa common to all six crops) was much smaller than the functional core (the set of metabolic functions common to all six crops): different crops are colonized by largely different bacteria, but those different bacteria tend to carry out a shared, overlapping set of functions. This is the same asymmetry we observe between our taxonomic (14.7%) and functional (7.8%) crop effects—different taxa, similar functional roles.

Matthews et al. [15] provide a complementary result. They compared two ways of predicting which bacteria would be present in a given plant’s rhizosphere: grouping bacteria by their functional traits (what genes/metabolic capabilities they carry) versus grouping them by 16S rRNA phylogeny (how closely related they are taxonomically). Functional grouping predicted rhizosphere membership more accurately (ANOSIM R = 0.44) than taxonomic grouping did (R = 0.31) across four plant species—that is, whether a bacterium ends up in a given plant’s rhizosphere depends more on what it can metabolically do than on which taxonomic group it belongs to. This supports the broader picture of functional redundancy: plants appear to recruit microbes for their functional capacity, and multiple, taxonomically unrelated bacteria can equally fill that role.

Wicaksono et al. [16] found that, at R^2^ = 50.5% for taxonomy, only 10.6% of genes were enriched simultaneously across all plant species examined, whereas 70.2% were species-specific; yet key functions—amino acid metabolism, carbohydrate metabolism, defense mechanisms—were enriched in the rhizosphere of all plants relative to soil. Our own data show the same pattern: pronounced taxonomic divergence between crops alongside a narrower, shared functional core. Zhou et al. [24] showed that a shared carbon source (decaying roots) can drive functional convergence (65–87% similarity between wheat and chickpea microbiomes with a shared detritusphere, versus 3–24% without it), and Li et al. [25] showed that diversity loss disrupts specific functions only in communities with initially low functional redundancy.

Redundancy in our data is incomplete: 36% of MetaCyc pathways and 45% of Kos remain significantly associated with crop even after normalization. Louca et al. [3] and Chen et al. [26] report that the degree of redundancy depends on function type: ‘broad’ central-metabolism functions are more redundant and resistant to taxonomic turnover than ‘narrow’, specialized functions. The three pathways robustly enriched in wheat across all three statistical methods (homolactic fermentation, peptidoglycan biosynthesis II, L-lysine biosynthesis II) belong to central metabolism—their persistent significance may reflect genuine differences in rhizosphere carbon/nitrogen turnover rather than noise.

Interpreting the functional profile requires acknowledging known PICRUSt2 limitations: Sun et al. [27] showed that metagenome prediction tools perform substantially worse outside the human microbiome; Matchado et al. [28] and Heidrich & Beule [29] argue that prediction from short 16S amplicons is ‘biologically limited’ and that NSTI does not always reliably reflect accuracy; Carter et al. [30] reported a median correlation of only ~0.20 between predicted and observed Kos. In our case, NSTI (median 0.0018, mean 0.216, 98.1% of ASVs < 2) is generally favorable, but the result remains a hypothesis about functional potential rather than a metagenomic measurement. Because 16S-based prediction can under-detect genuine functional differences as readily as it can inflate false positives, the smaller crop effect on the functional layer (relative to taxonomy) is consistent with functional redundancy but cannot be formally distinguished, on this evidence alone, from a ceiling on PICRUSt2 sensitivity; direct shotgun metagenomics or metatranscriptomics would be needed to arbitrate between these explanations.

The absence of a community-level effect does not imply that the inoculants failed to colonize the plant: *Bacillus subtilis* root colonization—chemotaxis toward root exudates followed by polysaccharide-induced biofilm formation—is well documented microscopically elsewhere [31,32]. Our 16S profiling, however, sampled the soil/rhizosphere pool rather than root tissue and cannot distinguish colonization from its absence; a negative result here therefore constrains only the soil-community signature of the inoculant, not root colonization itself. We did not directly measure any of the intermediate steps—root colonization, rhizosphere chemistry, or a transient community shift—so these data can rule out one candidate mechanism (community restructuring) but cannot establish what actually mediates the effect. Accordingly, the ‘integrative’ framing of this work refers to the joint measurement of the genome, microbiome, and phenotype, not a demonstrated causal chain between them.

Kong & Liu [4] propose that PGPR are often incapable of long-term colonization and that their effect may instead manifest through modification of the resident community rather than through establishment. Santoyo et al. [33] and Ribeiro et al. [34] similarly emphasize that inoculant establishment and persistence are highly variable and typically fail to translate laboratory advantages into a detectable community-level field effect. The non-monotonic dependence of the phenotypic effect on feeding frequency (peak at 2×, decline at 3×) is likewise consistent with a model of transient signaling action rather than gradual accumulation of an established colonization effect.

### 3.3. Strain Metabolism: The DNRA Pathway and the Biocontrol/Stress-Tolerance Trade-Off

The observed strain × frequency interaction—a substantial decline in 630 productivity at 4× application against relative stability in 1453—is plausibly explained by a single key gene: the presence of *nirD* in 1453 and its absence in 630, which determines the ability to complete the DNRA pathway (reduction of NO_2_^−^ to NH_4_^+^). Anaerobic microzones form around roots in the rhizosphere owing to oxygen consumption by microbial and root respiration [22]; under these conditions, strains carrying *narGHI* can use nitrate as an alternative electron acceptor [35]. In 1453, the NirB-NirD complex further reduces NO_2_^−^ to NH_4_^+^, retaining nitrogen in a plant-available form; in 630 this step is blocked, and accumulating nitrite may reach phytotoxic concentrations—NO_2_^−^ is known to inhibit photosynthesis and induce oxidative stress in plants [36]—an effect compounded by the increasing metabolic load of repeated application. Suharti et al. [37] note redundancy and modularity of membrane-associated dissimilatory nitrate reduction in *Bacillus* generally, consistent with the observed inter-strain variation in pathway completeness. This hypothesis requires direct experimental testing (measurement of NO_2_^−^/NH_4_^+^ in the rhizosphere, *nirD* complementation in 630) and, as it stands, remains a plausible but experimentally unconfirmed causal link.

A separate, complementary trade-off is apparent between productivity and stability: strain 1453, with stronger biocontrol genes (*bacE*, *sboA*, *srfAA*) and proteases (*nprE, nprB*), produces higher spike count and ear weight but with greater inter-replicate variability—possibly reflecting environmental dependence of lipopeptide production; Zhang et al. [38] report that *Bacillus* biocontrol efficacy via lipopeptides is strongly dependent on local environmental conditions and microbial context. Strain 630, with a more complete stress-tolerance regulon (*rsb*), produces a more modest but statistically more stable result. Strain 1453 is preferable for maximal productivity under controlled conditions; strain 630 is preferable for reliable performance under variable field conditions. Ji et al. [39] showed that co-inoculation with multiple *Bacillus* strains in the wheat rhizosphere can simultaneously provide biocontrol and growth promotion, consistent with the idea of a complementary consortium.

### 3.4. Study Limitations

**Figure 8:**
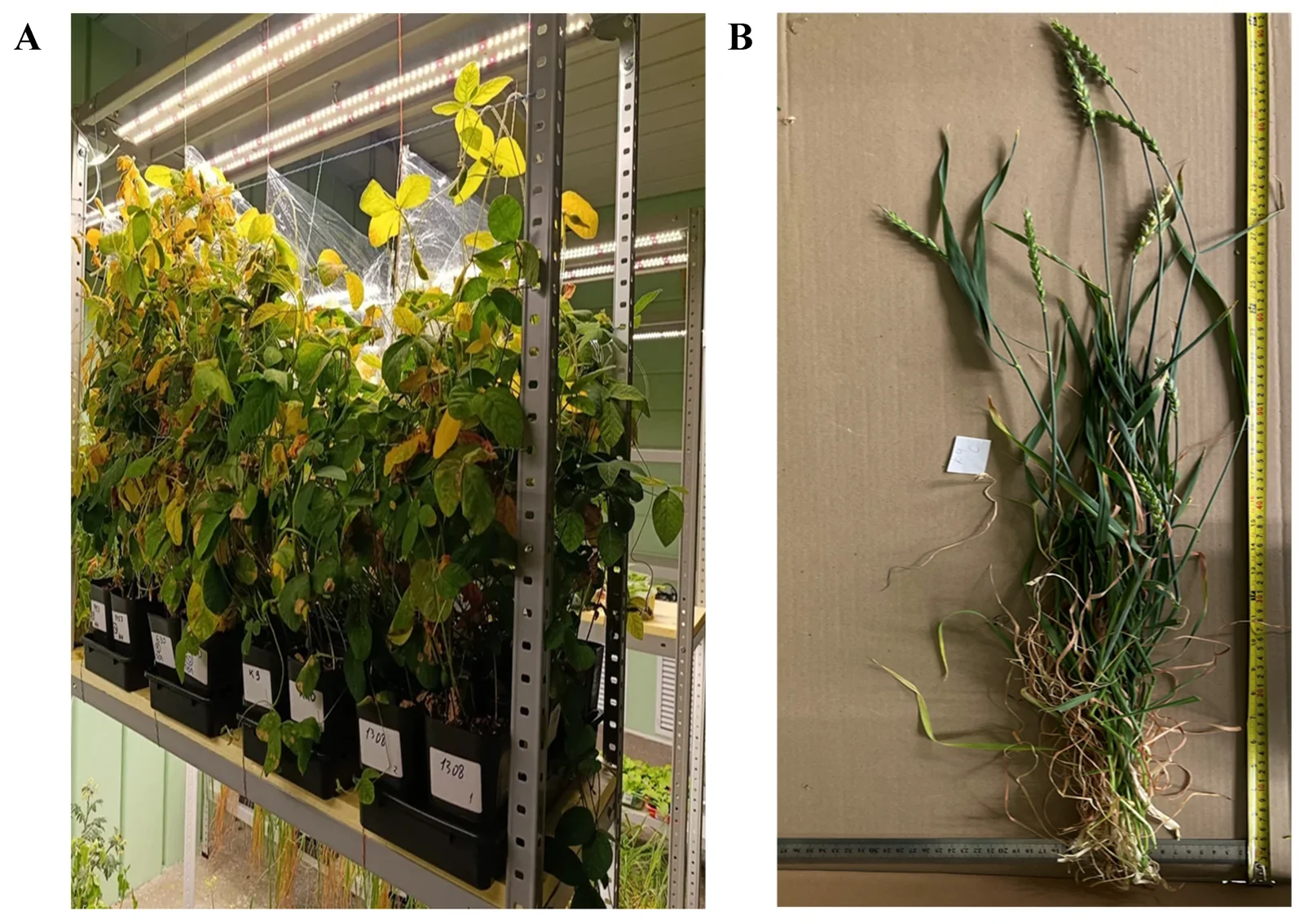
Soybean and wheat prior to harvest: pods filled and drying (**A**); wheat spike formed at the milk-ripe stage (**B**).

Several design features of this study warrant caution beyond the caveats already discussed alongside specific results (Section 2.2.2, Section 2.3.2 and Section 2.3.3). Sample size was small (3–4 replicates per treatment), limiting the power of the factorial ANOVA to detect smaller effects or higher-order interactions. Phenotype was recorded at a single time point, and wheat and soybean were sampled at different developmental stages (milk-ripe vs. pods filled and drying; Figure 8); species identity and developmental stage are therefore confounded within the crop factor and cannot be separated without stage-matched or time-series sampling. The pot experiment lacked heat-killed inoculum and carrier-only controls, so a contribution of non-biological matrix or handling effects (nutrients, osmolytes, pH) to the observed phenotypic gains cannot be excluded. Bacterial dose and root colonization were not quantified by CFU or qPCR across application counts, and no sterile-carrier control was run at 4×; the decline in strain 630 productivity at this level is therefore consistent with, but not proof of, nitrite phytotoxicity (Section 2.3.3), and could alternatively reflect a non-biological dose or handling effect.

Two further limitations concern data completeness rather than experimental design. Grain elemental composition (Table 4) was obtained from pooled composite samples without independent replicates and is interpreted descriptively only. Soybean phenotypic data are not analyzed here: replicate-level measurements were pooled by the collaborating team before transfer, reducing them to pseudoreplicates unsuitable for valid statistical inference. This was discovered only after receipt of the data. The corresponding wheat data were unaffected, as the original per-replicate records were independently recovered.

Finally, the two strains were preselected as the top two of 20 candidates in an earlier screen on the same crops; the effect magnitudes reported here (e.g., the +23.1% strain-1453 spike-count advantage) are therefore likely inflated relative to the true population-level performance of an arbitrary *Bacillus* PGPB candidate (winner’s-curse/regression-to-the-mean bias).

## 4. Materials and Methods

### 4.1. Experimental Design and Plant Material

A factorial pot experiment was conducted under greenhouse conditions (2025 season) on wheat (*Triticum aestivum*, cv. ‘Tobolskaya Stepnaya’) and soybean (*Glycine max*, cv. ‘Sibiryachka’). The two *Bacillus* strains used were isolated and deposited at the Laboratory of Molecular Genetics, ICBFM SB RAS, and were selected from a preliminary screen of 20 strains based on their effect on seedling germination and early growth (rolled-towel germination assay, wheat and soybean, 3- and 7-day measurements of germination energy, germination rate, and root/shoot length relative to an uninoculated control).

Two *Bacillus* strains—*B. halotolerans* 1453 and *B. pumilus* 630—were applied using two delivery methods: seed inoculation prior to sowing (denoted ‘In’, e.g., In1453, In630) and direct soil application of the suspension at sowing (denoted without the ‘In’ prefix, e.g., 1453, 630), combined with three application-frequency schemes: a single application at sowing only; the same application supplemented with an additional soil drench 2 weeks after sowing (suffix ‘x2′); and the same application supplemented with additional soil drenches at both 2 and 4 weeks after sowing (suffix ‘x3′). This yielded six treatment regimens per strain—for example, for strain 630: In630, In630x2, In630x3 (seed inoculation alone, with a 2-week drench, or with 2- and 4-week drenches, respectively) and 630, 630x2, 630x3 (soil drench at sowing alone, with a 2-week drench, or with 2- and 4-week drenches, respectively). Together with a bacteria-free control (‘CK’), this multifactorial pot experiment comprised 13 treatments per crop, with 3–4 biological replicates per treatment (the pot served as the independent experimental unit, with five plants per pot aggregated prior to measurement; pots were rotated to control for edge effects, with buffer pots placed along the perimeter). Phenotype was recorded at harvest: six traits for wheat (height, stem count, spike count, total weight, ear weight, green mass) and height for soybean. Individual replicate values were recorded separately.

#### 4.1.1. Inoculation Protocol

Bacterial cultures were grown at the Laboratory of Molecular Genetics, ICBFM SB RAS; the ready suspension was used for seed inoculation and substrate application. Bacterial cultures were grown in 50 mL Falcon tubes filled with 10 mL LB broth and were kept in a shaker at 200 rpm for 48 h, then diluted to adjust 10^8^ cfu/mL bacterial solutions with sterile, distilled water.

Seed inoculation was performed by the semi-dry biological seed-dressing method: 10 mL of bacterial preparation was added to the bottom of a round-bottom flask per 50 g of wheat seed or 100 g of soybean seed; the working solution was distributed over the flask walls, seeds were added, and the solution was applied to the seed surface by vigorous swirling of the flask until the walls were dry. Treated seeds were sown into the substrate in pots and placed in a germination chamber; after seedling emergence, pots were transferred to shelving in the phytotron room for subsequent watering and supplemental lighting. For the 2× and 3× application-count treatments, an additional 10 mL of the same bacterial suspension was applied directly to the substrate at 2 and 4 weeks after sowing, respectively.

#### 4.1.2. Growing Conditions

Plants were grown in a climate-controlled phytotron room: daytime temperature 22–24 °C, nighttime 20–22 °C, air humidity 50–60%, 18 h of supplemental lighting, full-spectrum light. The substrate was a peat mix of deacidified high-moor peat and agroperlite (3:1), with a pot volume of 1 L (five plants per pot). Wheat and soybean were grown from July to November (5 months); at harvest, wheat had not fully completed grain filling, but 60–80% of plants had flowered and formed a spike at the milk-ripe stage. At harvest, plants were photographed, above-ground biomass was cut, weighed, and dry matter was determined (Figure 8).

Plants received a single balanced mineral nutrient solution formulated for cereals and legumes (EC = 1.0 mS/cm): N-NH_4_ 11.4 ± 2.3 mg/L, N-NO_3_ 177.7 ± 21.3 mg/L, P_2_O_5_ 33.7 ± 6.7 mg/L, K 91.0 mg/L, Ca 90.0 mg/L, Mg 7.7 mg/L, Zn 0.18 mg/L, Cu 0.02 mg/L, Fe 0.49 mg/L, Mn 0.27 mg/L. The elemental ratio was held constant throughout the growing season; only solution concentration was adjusted based on plant diagnostics to avoid chlorosis.

### 4.2. 16S rRNA Sequencing and Processing

Rhizosphere samples were collected from 82 pots. Total DNA from 0.5 g soil was extracted using MagBeads FastDNA™ Kit for Soil (MP Biomedicals, LLC, Irvine, CA, USA) as recommended by the manufacturer. DNA quantity was estimated by Qubit 4.0 (Invitrogen/Life Technologies, Carlsbad, CA, USA).

The V3–V4 hypervariable region of the 16S rRNA gene was amplified with primers 341F (5′-CCTACGGGNGGCWGCAG-3′) and 805R (5′-GACTACHVGGGTATCTAATCC-3′) and sequenced on an Illumina MiSeq platform (2 × 301 bp paired-end). Primer sequences were trimmed with cutadapt [40] using a minimum post-trim length of 200 bp and quality threshold Q20. Sequence variants were inferred with DADA2 v1.22+ [41] using the following parameters: truncLen = c(240, 220), maxEE = c(2, 2), truncQ = 2, rm.phix = TRUE, pool = “pseudo” (pseudo-pooling for improved detection of rare variants), and consensus chimera removal. Merged sequences shorter than 400 bp were discarded. Taxonomy was assigned against the SILVA v138.2 reference database [42] using the assignTaxonomy function with reverse-complement matching prior to classification.

After quality filtering (filterAndTrim: truncLen = 240/220, maxEE = 2/2), 13 wheat samples with fewer than 1000 filtered reads were excluded, leaving 69 samples (28 wheat, 39 soy, and 2 bulk soil). Following denoising, merging, and chimera removal, the final read counts used for downstream analysis ranged from 155 to 25,288 per sample (wheat median = 402, soy median = 3062). A prevalence filter (presence in ≥ 2 samples) reduced the ASV count from 3267 to 1454. Plant-only samples (*n* = 67) were used for statistical analysis; 61 treated samples (excluding CK) were used for factorial models.

Read depth differed significantly between crops: wheat median 402 final reads (IQR 210–610) versus soy median 3062 (IQR 2029–5398), a 7.6-fold disparity (Mann–Whitney U, *p* = 1.3 × 10^−11^). To verify that the crop separation was not an artifact of this depth disparity, beta-diversity analyses were repeated after rarefaction to a uniform depth of 150 reads (retaining all 64 plant samples after exclusion of 3 outlier samples with >20,000 reads and elevated chloroplast contamination; see Section 2.3), and after alternative normalizations including CSS (metagenomeSeq; Paulson et al. [43]), DESeq2 variance-stabilizing transformation (VST; Love et al. [44]), and CLR transformation (Aitchison distance; Aitchison [45]). The crop effect remained significant under all methods (PERMANOVA *p* = 0.001), with rarefaction yielding the lowest depth correlation (ρ = 0.51) and the most conservative effect size (R^2^ = 0.047). This disparity was additionally addressed through compositional data analysis methods (Section 4.4).

### 4.3. Diversity and Differential Abundance Analysis

Alpha diversity indices (Observed ASVs, Chao1, Shannon, Simpson) were calculated on the unfiltered ASV table and compared across treatments using Kruskal–Wallis tests.

Chloroplast and mitochondrial sequences (9 ASVs) were removed prior to beta-diversity analyses, and three soy samples with exceptionally high read counts (21,000–25,000 reads) and elevated chloroplast contamination (2.8–38.3%) were excluded from the PCoA visualization as outliers.

Beta diversity was assessed using Bray–Curtis (abundance-weighted) and Jaccard (presence/absence) dissimilarities. Permutational multivariate analysis of variance (PERMANOVA; adonis2, 9999 permutations, vegan package; Oksanen et al. [46]) was performed under a factorial model: ~crop × strain × method × frequency. Results were visualized by principal coordinates analysis (PCoA). To assess the robustness of the crop separation to normalization choice and sequencing depth, PCoA and PERMANOVA were additionally performed after rarefaction to 150 reads, CSS normalization (metagenomeSeq; Paulson et al. [43]), DESeq2 VST [44], and CLR transformation with Aitchison distance [45]. Homogeneity of multivariate dispersions was tested with betadisper (999 permutations): dispersions differed significantly by crop (F = 10.07, *p* = 0.0007; wheat more dispersed than soy, mean distance-to-centroid 0.549 vs. 0.478) but not by strain, method, or frequency (all *p* > 0.05). To disentangle the crop effect from this dispersion heterogeneity and from the crop–depth correlation, PERMANOVA on Bray–Curtis dissimilarities was supplemented with covariate-adjusted PERMANOVA (read depth partialled in and out) and distance-based redundancy analysis (db-RDA, Condition (depth); less sensitive to dispersion heterogeneity than PERMANOVA), both on the cleaned dataset (*n* = 64, 1435 ASVs after chloroplast/mitochondria removal and outlier exclusion; 9999 permutations).

Differential abundance at the genus level was analyzed using three complementary methods: (i) ANCOM-BC2 [47] with structural zero detection and a sensitivity filter (q < 0.05); (ii) DESeq2 [42] with shrinkage estimators and independent hypothesis weighting (*p*adj < 0.05); and (iii) LEfSe [48] with LDA score threshold > 2.0 and *p* < 0.05. All analyses were performed on the prevalence-filtered ASV table aggregated to genus level.

### 4.4. PICRUSt2 Functional Prediction and Compositional Validation

Functional profiles were predicted using PICRUSt2 v2.6.x [49] against the PICRUSt2-SC reference database (GTDB r214, 27 870 genomes, EggNOG v2.1.2; Wright & Langille [50]). Predicted outputs comprised 519 MetaCyc pathways, 8186 KEGG Orthology (KO) entries, and 2678 Enzyme Commission (EC) numbers across all 69 samples. Prediction quality was assessed by the Nearest Sequenced Taxon Index (NSTI): mean = 0.216, median = 0.0018, with 98.1% of ASVs below the NSTI = 2.0 threshold.

Because relative abundance data are compositional, standard rank-based tests and Bray–Curtis dissimilarity can distort effect sizes [51]. The differential functional analysis was therefore validated by three independent methods of increasing compositional rigor:

1. Kruskal–Wallis tests on relative abundance (naïve approach, BH-adjusted)—the baseline against which compositional methods were compared.
2. ALDEx2 [52]—128 Monte Carlo instances, Welch’s *t*-test and Wilcoxon rank-sum test on CLR-transformed data with Benjamini–Hochberg correction; effect size threshold > 0.5.
3. ANCOM-BC2 [47] on raw pathway/KO/EC count tables, using its native sampling-fraction bias correction (not applied to CLR-transformed input), q < 0.05.

For ordination, PERMANOVA was performed on the Aitchison distance (Euclidean distance on CLR-transformed data; Aitchison [45]) with Bayesian zero replacement via the count-zero multiplicative method (cmultRepl/CZM, zCompositions package; Palarea-Albaladejo & Martín-Fernández [53]). Prevalence filtering (≥10% of samples) was applied prior to CLR transformation; features with >80% zeros were removed. The Aitchison PERMANOVA for the crop factor yielded R^2^ = 0.0783 (F = 5.01, *p* = 0.0001), with betadisper confirming homogeneity of multivariate dispersions (F = 1.88, *p* = 0.186). For comparison, the Bray–Curtis PERMANOVA on the same data yielded R^2^ = 0.0545 (F = 3.40, *p* = 0.0058), illustrating the compositional distortion inherent in relative-abundance-based distances.

A three-method comparison table (KW versus ALDEx2 versus ANCOM-BC2) was generated for pathways, Kos, and Ecs to quantify the reduction in false positives when moving from I to compositional methods.

### 4.5. Inoculant Strain Detection

Inoculant strains were traced in the 16S dataset by exact matching of ASV sequences against the inoculant full-length 16S rRNA gene sequences at 100% identity. Strain 630 was detected in 1 of 30 strain-630-treated samples (28 reads in the highest-depth sample at 25,288 reads); strain 1453 was not detected in any of 31 strain-1453-treated samples (closest match: 96.5% identity).

A binomial detection probability model was constructed as a function of read depth and true relative abundance: at the median wheat depth (402 reads), the probability of detecting a strain at 0.1% abundance was 33.1%, and at 0.01% abundance only 3.9%. An occupancy-detection model was fitted via maximum likelihood to estimate the true colonization probability (psi) and the per-read detection probability (r) compatible with the observed detections. A Monte Carlo simulation (10,000 iterations) was performed to assess the probability of observing 1/30 and 0/31 detections under different scenarios of true colonization (10%, 25%, 50%, 100% of samples) and abundance levels (0.01%, 0.1%, 0.5%).

### 4.6. Genome Mining of PGPB Genes

Genomes of strains 1453 (*B. halotolerans*) and 630 (*B. pumilus*) were analyzed using a combined three-method approach: (i) DIAMOND homology search [54], (ii) HMM profile search with HMMER3 [55], and (iii) Prokka annotation [56], against a curated database of 481 PGPB marker genes spanning 14 functional categories: phytohormones, ACC deaminase, nitrogen metabolism/fixation, siderophore/iron acquisition, phosphate solubilization, biocontrol/antimicrobial/lipopeptides, cell wall-degrading enzymes, volatile organic compounds, EPS/biofilm, quorum sensing/quenching, motility/chemotaxis/colonization, stress tolerance, heavy metal resistance, and secretion systems.

Gene status was classified hierarchically: confirmed (detected by all three methods, score = 3), likely (two methods, score = 2), putative (one method, score = 1), or absent (no method, score = 0). Category completeness was calculated as the percentage of genes present (any non-absent status) relative to the total genes in that category. Strong completeness (confirmed + likely only) was also tracked.

### 4.7. Plant Phenotype Statistical Analysis

Plant phenotype data were analyzed at two levels:

(i) Treatment-vs.-control comparisons. For count data (spike count), a Negative Binomial generalized linear model (NB-GLM; McCullagh & Nelder [57]) was used with a likelihood-ratio test against the null model and Benjamini–Hochberg correction. Overdispersion was assessed by the deviance-to-degrees-of-freedom ratio (deviance/df = 0.71), indicating no evidence of overdispersion (if anything, mild underdispersion); the NB model was nonetheless retained as a conservative choice. For ratio data (productive tillering = spike count/stem count), a binomial logit-GLM was fitted with quasi-binomial correction after detecting real overdispersion (Pearson χ^2^/df = 2.83). Each of the 12 inoculated treatments was compared to CK.
(ii) Factorial decomposition. A factorial ANOVA (2 × 2 × 3, excluding CK) was performed on individual replicate values using Type II sum of squares (for an unbalanced design). Main effects (strain, method, frequency) and the strain × frequency interaction were tested, with partial η^2^ as the effect size measure and Tukey HSD for post hoc pairwise comparisons. A one-way ANOVA (13 groups versus CK) with Dunnett’s test (Bonferroni-adjusted) and Tukey HSD was used as a complementary analysis to estimate effect magnitude independently.

Variance homogeneity was assessed by Levene’s test. The coefficient of variation (CV%) was computed per treatment × trait combination to evaluate phenotypic stability. Variance ratios (maximum/minimum CV) and Kruskal–Wallis tests on CV by factor level were used to identify sources of phenotypic instability.

### 4.8. Biochemical Characterization of Strains and Plants

The biochemical screening panel (phosphate solubilization, ammonium production, siderophore and gibberellin production, IAA production, and growth under osmotic stress) followed the protocols previously described [58]. Nutrient content (available nitrogen, phosphorus, potassium, and micronutrients) was determined as described in our earlier co-inoculation study [59].

### 4.9. Declaration of Generative AI Use

During the preparation of this manuscript, the authors used Biomni (Phylo, https://phylo.ai) for the purposes of bioinformatics data analysis and visualization, including PERMANOVA diagnostic analyses (betadisper, depth-adjusted models, db-RDA), systematic screening of bacterial strain genomes for nitrogen-cycle genes (BLASTp, HMM, tBLASTn), LEfSe and KEGG pathway functional profiling, and generation of corresponding figures and tables. The authors have reviewed and edited the output and take full responsibility for the content of this publication.

## 5. Conclusions

The genomic potential of PGPB inoculants predicts their phenotypic effect on the plant, even when inoculant establishment cannot be confirmed or ruled out by the methods available. This dissociation challenges the notion that PGPB act primarily through restructuring of the microbial community and instead supports a direct plant-microbe interaction model—in contrast to robust, biologically meaningful taxonomic differences between the wheat and soybean rhizosphere that are themselves independent of inoculation.

The DNRA pathway is a plausible, though experimentally unconfirmed, determinant of strain-specific dose response: the presence of *nirD* in 1453 enables complete reduction of nitrate to ammonium, whereas its absence in 630 may lead to nitrite accumulation and phytotoxicity at high application frequency. The trade-off between productivity, mediated by biocontrol (1453), and stability, mediated by stress tolerance (630), indicates that PGPB product design should consider not only the magnitude of the growth-promoting effect but also its robustness to variation in conditions.

## Figures and Tables

**Table 1:** PERMANOVA on Bray–Curtis dissimilarity (treated samples, *n* = 61).

Factor	R^2^	F	*p*-Value
Crop	0.1466	10.68	0.0001 ***
Strain	0.0161	1.17	0.2057
Application method	0.0109	0.79	0.7624
application count	0.0374	1.36	0.0581

*** (*p* = 0.0001).

**Table 2:** Differential functional analysis by crop.

Level	Total	KW (*p*adj < 0.05)	ALDEx2	ANCOM-BC2
MetaCyc pathways	519	187 (36%)	66 (13%)	79 (16%)
KOs	8186	3721 (47%)	527 (7%)	2292 (29%)
ECs	2678	856 (33%)	134 (5%)	—

**Table 3:** Strain-specific response to application count.

Trait	Strain	1×	2×	4×	Pattern
Stem count	630	31.7	22.8	19.4	Monotonic decline
Stem count	1453	25.7	21.8	23.8	Stable
Ear weight, g	630	9.4	11.0	7.4	2× optimum
Ear weight, g	1453	10.8	12.6	9.7	2× optimum, milder

**Table 4:** Grain elemental composition of wheat (mg/kg dry mass), control vs. treatments.

Treatment	P_2_O_5_, mg/kg	NH_4_, mg/kg	K, mg/kg	Ca, mg/kg	Mg, mg/kg
CK	16,174	118.7	76,713	8784	5387
I630	11,618	73.8	28,662	4446	2760
I630 × 2	9252	81.8	22,538	3049	2126
I630 × 3 *	12,980	95.8	—	—	—
630	9150	55.3	19,260	3457	1844
630 × 2	9231	84.7	22,308	4482	2287
630 × 3	10,353	67.0	24,881	17,174	1916
I1453	11,698	81.4	17,016	3110	1543
1453 × 2 *	12,498	86.2	—	—	—
1453 × 3 *	14,295	97.6	—	—	—

* K, Ca, and Mg were not available for treatments I630 × 3, L1453 × 2, and L1453 × 3 (marked —): these samples were lost during laboratory processing for technical reasons unrelated to treatment or biological effect.

**Table 5:** In vitro biochemical activity of strains 630 and 1453.

Trait	Strain 630	Strain 1453
Proline, µg/mL	70.0 ± 15.23	28.9 ± 8.11
Growth at 15% PEG, %	36.9 ± 2.93	15.4 ± 3.52
Phosphate solubilization, µg/mL	55.0 ± 4.84	95.3 ± 9.48
Siderophores, %	19.6 ± 5.45	35.6 ± 8.91
Gibberellins, mg/mL	0.80 ± 0.05	1.56 ± 0.12
IAA, µg/mL	0.92 ± 0.07	0.05 ± 0.04

**Table 6:** DNRA pathway genes: status in strains 1453 and 630.

Gene	1453	630	Function	Confidence
*narH*	confirmed	absent	Nitrate reductase, β-subunit (NO_3_ → NO_2_)	HIGH
*narJ*	confirmed	absent	Mo-cofactor assembly chaperone	HIGH
*narG*	confirmed	putative	Nitrate reductase, α-subunit	MODERATE
*nirB*	likely	putative	NADH-nitrite reductase (NO_2_ → NH_4_, DNRA)	MODERATE
*nirD*	likely	absent	Small subunit of the NirB-NirD complex	HIGH
*nasA*/*nasB*	confirmed	putative	Assimilatory pathway (NO_3_ → biomass)	MODERATE
*nrfA*	absent	absent	Periplasmic cytochrome c nitrite reductase (alternative DNRA route)	HIGH (absent)
*nrfH*	absent	absent	Cytochrome c quinol dehydrogenase (nrfA membrane anchor)	HIGH (absent)
*nirA*	weak	weak	Assimilatory nitrite reductase (N incorporation, not redox balancing)	LOW
*hmp*	putative	putative	Flavohemoglobin, NO detoxification (not nitrite reduction)	LOW-MODERATE
*norV*/*norW*	weak-putative	weak	Nitric oxide reductase, NO detoxification (not nitrite reduction)	LOW

## Data Availability

The genome assemblies of strains *B. pumilus* 630 and *B. halotolerans* 1453 are available in NCBI GenBank under accession numbers JBLUWR000000000 and JBWZYK000000000, respectively. The 16S rRNA amplicon sequencing raw reads generated in this study are being deposited in the NCBI Sequence Read Archive (SRA); accession numbers will be provided prior to publication. Phenotypic, biochemical, and statistical source data underlying the figures and tables are provided in the Supplementary Materials. Further data are available from the corresponding author upon reasonable request.

## Supplementary Materials

The following supporting information can be downloaded at: https://www.mdpi.com/article/10.3390/ijms27177873/s1.
