# Supplementary material for: Two Bacillus PGPB Strains in Wheat and Soybean: Wheat Growth Promotion Without Detectable Rhizosphere Microbiome Restructuring
Source: Int J Mol Sci. 2026 Sep 3;27(17):7873. doi: 10.3390/ijms27177873 (PMC13565908; doi:10.3390/ijms27177873)
Supplement: Supplementary file 1 [file ijms-27-07873-s001.zip › Table S1.anova_factorial_results.pdf]

crop	trait	effect	ss	df	F	p	partial_eta_sq	significant
wheat	height	C(strain)	0.1653	1	1.0096	0.32361394907625757	0.0348	False
wheat	height	C(method)	0.2572	1	1.5712	0.22039770654210192	0.0531	False
wheat	height	C(frequency)	0.3895	2	1.1894	0.31931511754116887	0.0783	False
wheat	height	C(strain):C(method)	0.2987	1	1.8243	0.1876176676186437	0.0612	False
wheat	height	C(strain):C(frequency)	0.8207	2	2.5063	0.09970404995247936	0.1518	False
wheat	height	C(method):C(frequency)	0.4584	2	1.4	0.26332393314689	0.0909	False
wheat	height	C(strain):C(method):C(frequency)	0.0131	2	0.0399	0.960937780229273	0.0028	False
wheat	height	Residual	4.5843	28			0.5	False
wheat	stems	C(strain)	1.225	1	0.069	0.7946506386144695	0.0025	False
wheat	stems	C(method)	30.625	1	1.7262	0.19955776899526395	0.0581	False
wheat	stems	C(frequency)	389.5042	2	10.9775	0.0003020745943134205	0.4395	True
wheat	stems	C(strain):C(method)	18.225	1	1.0273	0.3194777599725167	0.0354	False
wheat	stems	C(strain):C(frequency)	186.3375	2	5.2516	0.01156808649039831	0.2728	True
wheat	stems	C(method):C(frequency)	1.1042	2	0.0311	0.9693937473232982	0.0022	False
wheat	stems	C(strain):C(method):C(frequency)	49.0042	2	1.3811	0.26789920174444964	0.0898	False
wheat	stems	Residual	496.75	28			0.5	False
wheat	spikes	C(strain)	62.5	1	7.1869	0.012168964174141003	0.2042	True
wheat	spikes	C(method)	52.9	1	6.083	0.020035400025857204	0.1785	True
wheat	spikes	C(frequency)	176.9833	2	10.1756	0.0004769825691044134	0.4209	True
wheat	spikes	C(strain):C(method)	0.9	1	0.1035	0.7500694967187632	0.0037	False
wheat	spikes	C(strain):C(frequency)	78.5833	2	4.5181	0.019926452433080086	0.244	True
wheat	spikes	C(method):C(frequency)	24.2667	2	1.3952	0.2644809196152864	0.0906	False
wheat	spikes	C(strain):C(method):C(frequency)	24.2667	2	1.3952	0.26448091961528597	0.0906	False
wheat	spikes	Residual	243.5	28			0.5	False
wheat	total_weight	C(strain)	20.164	1	0.3219	0.5749811653245112	0.0114	False
wheat	total_weight	C(method)	332.237	1	5.3041	0.02891580109708678	0.1593	True
wheat	total_weight	C(frequency)	663.7892	2	5.2986	0.011179562319954562	0.2746	True
wheat	total_weight	C(strain):C(method)	37.481	1	0.5984	0.4456808787670773	0.0209	False
wheat	total_weight	C(strain):C(frequency)	158.5924	2	1.2659	0.2976189607547974	0.0829	False

wheat	total_weight	C(method):C(frequency)	18.3322	2	0.1463	0.8645246745420269	0.0103	False
wheat	total_weight	C(strain):C(method):C(frequency)	68.7402	2	0.5487	0.5837800780134077	0.0377	False
wheat	total_weight	Residual	1753.8618	28			0.5	False
wheat	ear_weight	C(strain)	34.1141	1	5.7498	0.02340133099649987	0.1704	True
wheat	ear_weight	C(method)	7.208	1	1.2149	0.27975209225191233	0.0416	False
wheat	ear_weight	C(frequency)	72.1155	2	6.0774	0.006425301136228739	0.3027	True
wheat	ear_weight	C(strain):C(method)	5.7305	1	0.9659	0.33413359795107556	0.0333	False
wheat	ear_weight	C(strain):C(frequency)	1.5799	2	0.1331	0.875888397476252	0.0094	False
wheat	ear_weight	C(method):C(frequency)	28.1941	2	2.376	0.11139908603170211	0.1451	False
wheat	ear_weight	C(strain):C(method):C(frequency)	29.3733	2	2.4754	0.10235275921986535	0.1502	False
wheat	ear_weight	Residual	166.1269	28			0.5	False
wheat	green_mass	C(strain)	106.7329	1	1.4635	0.23649212032255815	0.0497	False
wheat	green_mass	C(method)	241.5723	1	3.3123	0.07946806080635763	0.1058	False
wheat	green_mass	C(frequency)	779.8357	2	5.3464	0.010799126924055467	0.2764	True
wheat	green_mass	C(strain):C(method)	13.9004	1	0.1906	0.665768257628451	0.0068	False
wheat	green_mass	C(strain):C(frequency)	129.6609	2	0.8889	0.4223802143551688	0.0597	False
wheat	green_mass	C(method):C(frequency)	91.505	2	0.6273	0.5413488436540103	0.0429	False
wheat	green_mass	C(strain):C(method):C(frequency)	32.0983	2	0.2201	0.8038457091396142	0.0155	False
wheat	green_mass	Residual	2042.0638	28			0.5	False
soy	height	C(strain)	1.5607	1	0.6845	0.4150319567340039	0.0239	False
soy	height	C(method)	3.1014	1	1.3603	0.25333164598644176	0.0463	False
soy	height	C(frequency)	2.9004	2	0.636	0.5368650812187971	0.0435	False
soy	height	C(strain):C(method)	6.5958	1	2.8928	0.10005668170477577	0.0936	False
soy	height	C(strain):C(frequency)	7.5319	2	1.6517	0.20985800889317802	0.1055	False
soy	height	C(method):C(frequency)	4.4874	2	0.9841	0.38634443956555714	0.0657	False
soy	height	C(strain):C(method):C(frequency)	3.5901	2	0.7873	0.46489183738894685	0.0532	False
soy	height	Residual	63.841	28			0.5	False
