# Supplementary material for: Two Bacillus PGPB Strains in Wheat and Soybean: Wheat Growth Promotion Without Detectable Rhizosphere Microbiome Restructuring
Source: Int J Mol Sci. 2026 Sep 3;27(17):7873. doi: 10.3390/ijms27177873 (PMC13565908; doi:10.3390/ijms27177873)
Supplement: Supplementary file 1 [file ijms-27-07873-s001.zip › Table S3. Curated gene-level hypotheses linking genomic differences to phenotypic outcomes (H1–H6)..pdf]

Hypothesis	Genes	Proposed mechanism	Phenotypic link
H1: DNRA-mediated N conservation	*narH, narJ, narG, nasA, nasB, nirD*	$NO_3^- \rightarrow NO_2^- \rightarrow NH_4^+$ via DNRA; complete in 1453, truncated in 630 (no *nirD*)	1453 +23.1% spikes, +20.4% ear weight vs. 630
H2: Biocontrol lipopeptides	*bacE, sboA, srfAA*	Bacilysin/subtilosin/surfactin — pathogen suppression, possible phytotoxicity at high titre	1453 higher spike/ear-weight CV (24.4%/25.5% vs. 20.6%/16.4%)
H3: Extracellular proteases	*nprE, nprB*	Organic-N mineralization → plant-available N	1453 unique; +23.1% spikes, +20.4% ear weight
H4: General stress tolerance	*rsbU, rsbV, rsbW, cspC, otsB*	σ-B regulon, cold-shock, osmoprotection → colonization consistency	630 lower CV (ear weight 16.4% vs. 25.5%; spikes 20.6% vs. 24.4%)
H5: Phosphate solubilization	*phyA, pqqD*	Phytase/PQQ-glucose dehydrogenase → P availability	Discrepant: 630 has genomic edge, but no total-weight advantage (−2.9% vs. 1453)
H6: Motility-dependent method response	*fimA*	Type IV pili → root surface attachment	Discrepant: gene unique to 630, but no strain×method interaction detected
